# Modernizing a National Electronic Health Record: a Learning Health Care System Approach

**DOI:** 10.1007/s11606-023-08334-w

**Published:** 2023-10-05

**Authors:** David Atkins, Carolyn Clancy, Shereef Elnahal

**Affiliations:** 1 Health Services Research and Development Service, Office of Research and Development (14RD), Veterans Health Administration, 810 Vermont Avenue NW, Washington, DC 20420 USA; 2 VHA Office of Discovery, Education and Affiliate Networks (14), Veterans Health Administration, 810 Vermont Ave. NW, Washington, DC 20420 USA; 3 Office of the Under Secretary for Health, Veterans Health Administration, Washington, DC USA

Never before has the need for a modernized electronic record supporting Veterans’ health been clearer. A common, interoperable platform that seamlessly connects records from military service to VA is essential to our mission of caring for those who have served in our nation’s military and for their families, caregivers, and survivors. Such a platform is critical to VA becoming a high-reliability organization that ensures enterprise-wide consistency in clinical practice. It is also fundamental to our role as a learning health system that informs US health care overall.

As seen in the private sector, electronic health record (EHR) deployment, even under the best of circumstances, is rarely seamless. The fact that VA began its transition to a new system 7 months into a global pandemic added an unprecedented layer of complexity. Additionally, unlike deployments in the private sector, this transition occurred under the public microscope and with a high level of transparency. Together, these two recurring components of quality improvement—accountability and transparency—have been foundational to VA’s history as a learning health care system.^1^

Another chapter in that history, as noted by several papers, has been the evolution of the VA research program to promote actionable inquiry on ongoing changes in VA healthcare. This type of inquiry can help assure that successive EHR deployments are enhanced through shared learning and subsequent improvements. The nation’s emergence from the pandemic, as well as new priorities such as the PACT Act,^1^ makes our commitment to being a learning health care system even more important.

As further context for these, as well as other articles, this commentary provides a synopsis of VA’s longstanding EHR work, lessons learned from initial deployment, and research questions that VA is poised to address.

## Va’s Stake in the Ground

VA’s development of an EHR dates back to the 1970s; the journey over the subsequent 40 years is emblematic of VA’s forward-leaning approach to Veteran care and longstanding history in innovation. What began as a series of collaborations among VA, the US Public Health Service, the Department of Defense (DoD), and the Indian Health Service culminated in the 1978 launch of initial modules of an electronic information system at 20 VA medical centers.

Less than 20 years later, then VA Under Secretary for Health Dr. Kenneth Kizer committed the Department to implementation across the national VA enterprise, christening the system “VistA” (Veterans Health Information System and Technology Architecture). Because the launch coincided with VA’s expansion to include both outpatient and inpatient care, VistA encompassed both modes of delivery from the outset.

Since then, a common national EHR platform has allowed VA to share patient information efficiently, track system performance, and drive quality improvement. Notably, VistA/Computerized Patient Record System (CPRS) has been popular with VA clinicians, likely reflecting their extensive involvement in its development (e.g., the system includes an interface that is customizable to local needs).

However, as VA’s EHR began to take hold nationwide, the same capability that allowed individual facilities to customize worked against the ability to scale new innovations in health information technology and promote common clinical care practices. In other words, VA did not have one medical record—it had over 130 versions of a common platform reflecting local customization.

Despite a decade of collaboration, the goal of creating a common interoperable record between VA and DoD remained out of reach. Consequently, in 2017 VA announced the Department would transition from VistA/CPRS to a commercial off-the-shelf EHR platform, selecting the same system DoD had earlier competitively selected for its 490 health care facilities in 2015.

## The Electronic Road not Traveled

As outlined in the agreement, VA’s transition toward a new EHR at its 1454 medical centers and outpatient clinics would be the nation’s largest and most ambitious implementation. Over a 10-year phased rollout, the implementation would seek to address myriad, inter-related issues that also challenged private sector health care.

For example, a well-known implementation challenge is the potential for health information systems to cause errors, not just prevent them.^2^ These issues can involve problems in software design and clinician confusion.

Furthermore, while the benefits of moving from a paper-based system to EHR were established more than a quarter century ago, the evidence-base regarding certain critical areas has been slower to develop. In its 1997 report, the Institute of Medicine discussed benefits such as improving quality, productivity, research, and data confidentiality.^3^ Since then, most research has focused on documenting the impact of converting to a new record. Issues such as unintended effects on workload, communication among providers, and efficiency as well as the doctor-patient relationship and quality of care^4,5^ have yet to be fully explored.

Moreover, research has yet to document the challenge of ensuring a safe and satisfying transition from one EHR to another. Since no vendor has ever undertaken a task of this size, it was assumed there would be numerous unanswered questions about implementing a new EHR in VA’s variety of environments. (Some of VA’s work addressing these issues is presented in the article about the research consortium called PROVEN Coordinating Hub to Promote Research Optimizing Veteran-centric EHR Networks.)

In anticipation of these issues, and with a goal of addressing the research gaps, the Health Services Research and Development Service within VA’s Office of Research and Development convened a meeting early in the process in 2019 with the VA Office of Electronic Health Record Modernization. Together, these offices mapped out possible areas where research could help with what was anticipated to be a large, complex, and challenging task.

## Early Deployment

In October 2020, VA began implementation of its new EHR at several sites. At the same time, the global pandemic was in its relative infancy with cases surging, health care workers experiencing extreme stresses, and supply chains being disrupted. With both VA employees and EHR trainers also becoming infected, implementation assistance was sharply limited.

In addition to training, other challenges emerged, such as lost consults and incomplete data migration. Many of these issues were documented in Office of Inspector General and General Accounting Office reports and through Congressional oversight. At the same time, VA maintained a level of transparency that enabled it to quickly respond to these and other concerns by conducting a patient safety review. VA also initiated a “sprint” team to assess required fixes to patient safety issues. While the platform was adapted to needs based on recommendations by a network of VA clinical councils, the scale of implementation meant it could not be further customized based on experience of frontline clinicians as it was rolled out. As a result, VA announced a strategic “reset” in early 2023 that indefinitely paused further new implementation until initial implementation issues could be examined and addressed.

## Moving Forward: The Role of Research

As this special issue of *JGIM* demonstrates, VA’s research program includes nationally recognized experts on EHRs and patient safety. Moving forward, their expertise, in collaboration with other VA national program offices and frontline staff, will enable us to leverage lessons learned from initial deployment.

To start, there are three specific areas where the type of deep analysis allowed by research can contribute to findings of traditional Patient Safety and IT sprint teams. First, we need to deepen our understanding of patient safety issues by developing a more systematic set of safety monitors and metrics that can be assessed proactively, rather than reactively.

Second, we need a stronger assessment of user experience and the role of training, with the aim of identifying the most valuable elements of training. This assessment must include identifying reliable approaches to minimize impacts on clinicians’ resilience. Already, researchers have begun to articulate mitigating factors, including the impact of local leadership engagement.

Third, we need to fully understand the implementation’s effect on provider productivity. This will help sites deploying the EHR plan for additional support during initial startup while clinicians spend time adjusting to the new system.

Beyond research, we must also recognize that new technologies and trends—artificial intelligence, patient-generated data, predictive analytics, and precision medicine to name a few—mean we must remain tightly focused on the EHR’s primary function: helping clinicians guide patients to the right treatment at the right time. Developing and scaling these innovations will be much easier with a single, consistent EHR platform. Moreover, innovations may help address “death by 1,000 clicks,” thereby allowing clinicians to spend more time listening to patients and less time at their keyboard. Finally, having more data will unlock new research avenues.

While the need for a new EHR is clear, and the potential advantages compelling, our first commitment is to Veterans and the dedicated clinicians caring for them. Advances in information and communications technologies demand that EHRs develop capabilities that, at a minimum, do not preclude continued learning and improvement.

The initial launch of a new EHR during COVID-19 frequently made it feel as if we were still building the plane while flying it. Today, as we emerge from a pandemic that revealed health inequities and left many physicians feeling burned out, we have recommitted to getting things right for patients and providers before proceeding further. As the papers in the special edition show, VA is actively using lessons learned from initial EHRM deployment to ensure that future rollout is safe, secure, and seamless for all.

## Footnotes

1 The Sergeant First Class Heath Robinson Honoring Our Promise to Address Comprehensive Toxins Act of 2022, or PACT Act. A common interoperable platform that seamlessly connects records from military service to VA is essential for implementing the PACT Act which will provide care for conditions that may be due to deployment-related toxic exposures and expanding our understanding of the impact of those exposures.
